# Hyperbaric Oxygenation as Adjunctive Therapy in the Treatment of Sudden Sensorineural Hearing Loss

**DOI:** 10.3390/ijms21228588

**Published:** 2020-11-14

**Authors:** Dorota Olex-Zarychta

**Affiliations:** Institute of Sport Sciences, Academy of Physical Education in Katowice, 40-065 Katowice, Poland; d.olex@awf.katowice.pl

**Keywords:** sensorineural hearing loss, hyperbaric oxygenation, adjunctive therapy

## Abstract

Sudden sensorineural hearing loss seems to become a serious social health problem in modern societies. According to the World Health Organization (WHO) reports, adult-onset sensorineural hearing loss is found to be one of the leading diseases at the global level, especially in high-income countries, and is foreseen to move up from the 14th to 7th leading cause of the global burden of diseases by the year 2030. Although the direct mortality rate of this disease is very low, its influence on quality of life is huge; that is the reason why the implementation of the most effective and the safest therapies for the patient is crucial for minimizing the risk of complications and adverse reactions to treatment. The aim of this paper is to present hyperbaric oxygen therapy (HBOT) as a medical procedure useful in the treatment of sudden sensorineural hearing loss as adjunctive therapy of high efficacy. This paper focuses on the molecular mechanisms of action and clinical effectiveness of HBOT in the treatment of idiopathic sudden deafness, taking into consideration both the benefits and potential risks of its implementation.

## 1. Introduction

Clinical hyperbaric oxygen therapy (HBOT) is defined as placing a patient’s entire body in an increased pressure environment and having that patient inhale 100% oxygen for their specific diagnosis, for a defined period of time per treatment [1]. HBOT can be used as the leading or a complementary therapy to address several medical problems. HBOT is undertaken in Europe on the basis of the indications of the European Committee of Hyperbaric Medicine (ECHM). Actual indications for the medical use of HBOT in Europe were established during the ECHM Consensus Conference in 2016, taking into account three different types of recommendations for using HBOT as a medical procedure.

A Type I recommendation is where HBOT is strongly recommended, because this approach guarantees a positive treatment effect. Recommendations include decompression illness and CO poisoning, prevention and treatment of osteoradionecrosis (mandible), soft tissue radionecrosis (cystitis and proctitis), gas embolism, open fractures with a crush injury, anaerobic or mixed bacterial infections, and sudden sensorineural hearing loss (SSNHL). Recommendations and standards are supported by strong evidence of beneficial action based on at least two concordant, large, double-blind, randomized controlled trials, with no or only weak methodological bias.

A Type II recommendation is when HBOT is recommended because of its positive therapeutic effect. Recommendations include diabetic foot lesions, femoral head necrosis, compromised skin grafts and musculo-cutaneous flaps, a crush injury without a fracture, osteoradionecrosis (bones other than the mandible), radio-induced lesions of soft tissues (other than cystitis and proctitis), ischemic ulcers, refractory chronic osteomyelitis, and 2nd-degree burns over more than 20% of the body surface area. Recommendations or guidelines are supported by evidence of beneficial action based on double-blind, randomized controlled trials, but with some methodological bias, such as concerning only small samples or only a single study. 

A Type III recommendation is when choosing HBOT may be optional, such as for radio-induced lesions, post-vascular procedure reperfusion syndrome and limb replantation, and selected non-healing wounds secondary to systemic processes. Recommendations are supported by weak evidence of beneficial action based only on uncontrolled studies-a historical control group, a cohort study, etc. [2].

Sensorineural hearing loss seems to become a serious social health problem in modern societies. According to the World Health Organization (WHO) reports, sensorineural hearing loss is one of the leading diseases at the global level, especially in high-income countries, and is foreseen to move up from the 14th to 7th leading cause of the global burden of diseases by the year 2030 [3]. Although the direct mortality rate of this disease is very low, its influence on quality of life is significant; that is, the reason why the implementation of the most effective and the safest therapies for the patient is crucial for minimizing the risk of complications and adverse reactions to treatment. The aim of this paper is to present HBOT as a medical procedure useful in the treatment of sudden sensorineural hearing loss (SSNHL) as an adjunctive therapy of high efficacy. This paper focuses on the mechanisms of action and clinical usefulness of HBOT in the treatment of idiopathic sudden deafness, taking into consideration both the benefits and potential risks of its implementation.

## 2. Physiology of Hyperbaric Oxygenation in Human 

For oxygenation in hyperbaric conditions, pressure is expressed as multiples of the atmospheric pressure measured at sea level, which is 1 atmosphere absolute (1 ATA). In normobaric conditions at sea level, most of the oxygen in the blood is in a complex with the hemoglobin from the red blood cells (98%), with just a tiny amount of oxygen actually dissolved in the plasma (0.3 mL oxygen/100 mL). Assuming normal perfusion, tissues at rest extract about 6 mL of oxygen per 100 mL of blood. Breathing with pure oxygen in hyperbaric conditions leads to an increase in oxygen partial pressure in the lungs, and thus a significant increase in its concentration in the plasma on the basis of physical dissolution in amounts of up to 20 times higher than at ambient pressure. Hyperbaric oxygenation increases the oxidative capacity of the blood serum. Breathing 100% oxygen under hyperbaric conditions (f. ex. 2.5 ATA) leads to increased amounts of oxygen in blood serum to about 5 mL/100 mL. During HBOT, the pressure is raised to 3 ATA and the amount of oxygen delivered with the blood increases from 10 to 15 times above the norm, which gives a quantity sufficient for life support even in the case of absence of hemoglobin [4]. In hyperbaric conditions, the amount of oxygen that is dissolved in the blood plasma is sufficient to meet resting requirements. Administering 100% oxygen at 3 ATA, the dissolved oxygen content is approximately 6 mL per 100 mL, while in normobaric conditions breathing pure oxygen increases the amount of oxygen dissolved in the blood only from 0.3 to 1.5 mL per 100 mL. Breathing 100% oxygen in hyperbaric conditions above 1.4 ATA contributes to a significant increase in the diffusion radius of the oxygen from the capillaries to the surrounding tissues. At 3 ATA, its plasma pressure can be as high as 2000 mmHg, which increases the diffusion of oxygen to the tissues four times on the arterial side, and twice on the venous side of the capillary blood circulation [5]. Breathing 100% oxygen under normobaric conditions (1 ATA) is not qualified as HBOT, since it increases the amount of oxygen dissolved in the blood serum only about 2 mL/100 mL [6]. HBOT also causes an increase in the elasticity of erythrocytes and reduces the viscosity of blood, thus improving microcirculation [6,7]. Inhaling the oxygen at 3 ATA increases the partial pressure of the oxygen in the blood to 200 kPa and more, which leads to an increase in the oxygen concentration in arterial blood of about 6.6–6.8 mL oxygen/100 mL [4].

The influence of HBOT on organs and tissues is diverse. In conditions of high pressure and high partial concentrations, the oxygen becomes a drug exerting many important phenomena in a patient’s body, the most important of which is metabolism [5]. Hyperbaric oxygen is proven to have a beneficial influence on local hypoxia in tissues. Proper oxygen tension is essential for angiogenesis because it is a prerequisite for the formation of the collagen matrix [8]. The use of HBOT decreases the ability of neutrophils to adhere to the vessel walls, thus reducing endothelial damage, and allowing vasoconstriction of vessels in areas with a normal oxygen concentration without changes in the circulation in areas with impaired flow, restoration of collagen production and fibroblast growth, stimulation of peroxide dismutase production, and storage of adenosine-triphosphate (ATP) in cell membranes. This has an influence on the reduction of edema in tissues, limitation of some forms of immune response, stimulation of osteoclasts activity, capillary proliferation, inhibition of production the surfactant in the lungs, and blockage of lipid peroxidation in carbon monoxide (CO) poisoning and its accelerated removal from hemoglobin [7]. In conditions caused by the formation of gas (f ex. in cases of decompression sickness), through increasing the pressure HBOT causes a reduction in the volume of the inert gas bubbles in the blood vessels and tissues. At 2.8 ATA, the bubble volume is reduced by almost two thirds. Treatment with HBOT serves to decrease the bubble size, thus reducing pain and restoring blood flow, while also correcting tissue hypoxia and inducing a large inert gas gradient to expedite washout. Hyperbaric oxygenation hastens additionally the dissolution of the gas bubbles by replacing the inert gas in the bubble with oxygen (metabolized rapidly by the tissues) and prevents the formation of new bubbles [9]. 

The administration of pure hyperbaric oxygen extends the leukocyte antibacterial activity. It increases the rate of killing of some common bacteria and phagocytes. HBOT is bactericidal for certain anaerobes, including *Clostridium perfringens*, and research showed that it is bacteriostatic for some species of *Escherichia* and *Pseudomonas* [10]. The action of HBOT on anaerobes is based on the production of free radicals, such as superoxide, dismutase, catalase, and peroxidase. Many different clostridial exotoxins have been identified so far, and the most prevalent is alphatoxine (phospholipase C), which is hemolytic and tissue necrotizing, as well as thetatoxine, which is responsible for vascular injury and in consequence for acceleration of tissue necrosis. HBOT blocks the production of alphatoxine and thetatoxine and acts as inhibitors of bacterial growth [11]. Hyperbaric oxygen is documented to reduce ischemia/reperfusion injury in a number of different experimental models. Mechanisms responsible for the beneficial effect of HBOT in the treatment of ischemia/reperfusion injury involve suppression of neutrophil–endothelial adhesion [12]. The effect of inhibiting leukocyte adhesion to the endothelium is present after HBOT, and it leads to improvement in microcirculation [13]. Huang and Obenaus [14] reported the neuroprotective effect of HBOT in an animal model. They observed anti-inflammatory effects and an improvement in tissue oxidation, as well as inhibition of mitochondria-associated apoptosis. There is little available data on the influence of HBOT on the motor control and neurocognitive functioning of humans; however, a positive effect of HBOT on sensorimotor functions in mammals was suggested in some previous research. A positive effect of HBOT on simple reaction time is showed in the research of Olex-Zarychta [15], where after 15 sessions at 2.5 ATA the reaction time of the patient visibly improved, and 3 weeks after the last session slightly decreased, but still exceeded the output level. Research of Kenneth and Stoller [16] presented the beneficial effects of HBOT on human psychomotor functions, but the effects of therapy were transient; maintaining the positive effect of HBOT required repeated administering. The potential mechanism that may underline the effectiveness of hyperbaric oxygenation on neurocognitive functions of humans is still not clear. It is supposed that HBOT leads to a higher amount of oxygen available to the brain and, therefore, it results in an increase in the cognitive processing of the human. Appropriate HBOT exposure results in an increase in the oxygen content of arterial blood. This increase in oxygen and the subsequent increase in the oxygen diffusion gradient between the blood and tissues has been termed the primary effect of HBOT therapy. A secondary effect of HBOT therapy is a reduction in the inflammatory response due to a vasoconstriction mediated by the oxygen [17]. Such therapy can indirectly result in an increased ability to repair blood vessels, improvement in the circulation in brain tissue, and may increase the ability of vascular endothelium toward axon regeneration or nitrogen synthesis [18].

## 3. The Use of Hyperbaric Oxygenation in Medicine

Hyperbaric oxygenation as a medical procedure involves a patient inhaling pure oxygen using a pressure of 2 to 3 ATA, which is provided by properly constructed pressure chambers. Breathing 100% oxygen at ambient pressure of 1 ATA, or displaying only parts of the body at 100% oxygen, should not be qualified as HBOT [6,7]. Current knowledge indicates that the pressure in a hyperbaric chamber during HBOT should be at least 1.4 ATA [4]. According to ECHM standards and guidelines, one typical session of HBOT in a multi-site chamber lasts about 90 min and consists of three main phases. The first phase lasts about 10 min and contains air compression to the pressure of 2.0–2.5 ATA (equivalent to the depth of 10–15 m under water) and lasts 70 min. During the second phase, the patient breathes pure oxygen through a mask three times for 20 min, taking two air breaks (5 min each) with the mask off. The last phase of the session is decompression [6]. The procedure for administering pure oxygen in an intermittent way is to avoid symptoms of oxygen toxicity. In almost all centers of hyperbaric medicine around the world, 100% oxygen is administered in hyperbaric conditions of 2–3 ATA in an intermittent way, in a time not exceeding 1 h. 

Clinically, HBOT is highly efficacious in the treatment of several conditions spanning a broad pathological range [19]. Actual European indications for the medical use of HBOT were established during the ECHM Consensus Conference in 2016, taking into account three types of recommendations for using HBOT as a medical procedure—from strongly recommended to optional use, depending on the strength of the evidence of its beneficial action [2]. In the USA, such indications are prepared by the Undersea and Hyperbaric Medical Society (UHMS). Both the ECHM and UHMS create and update lists of indications for treatment with hyperbaric oxygen every few years. Differences between the actual lists include the lack of indications for SSNHL in UHMS recommendations (present on the ECHM list) and the lack of indications for a state of extremely high blood loss on the ECHM list (present on the UHMS list). HBOT is also used in treatment of diseases not included in the official lists of indications, although further studies need to confirm the efficacy of such an approach [5]. Some authors indicate the neurotherapeutic effects of hyperbaric oxygen in traumatic brain injuries, in the light of recent evidence for HBOT efficacy in brain repair [20]. Some observations and experiments indicated HBOT as a potent means of delivering to the brain sufficient oxygen needed for activation of neuroplasticity and restoration of impaired brain functions [21]. However, there is no strong evidence of the effectiveness of HBOT in neurodegenerative illnesses so far. The lack of a clear and confirmed clinical effect of HBOT is why in the 2016 recommendations it could not be used in autism spectrum disorders, placental insufficiency, multiple sclerosis, cerebral palsy, and acute phase of stroke [2]. Complete contraindications to HBOT are untreated pneumothorax and chemotherapy. Relative contraindications to oxygenation in hyperbaric conditions are upper respiratory tract infections, emphysema with CO_2_ retention, asymptomatic air cysts or blebs in the lungs, high body temperature, pregnancy, claustrophobia, low seizure threshold, bleomycin therapy, and a history of thoracic or ear surgery [2,7].

## 4. HBOT in the Treatment of Sudden Sensorineural Hearing Loss

Sudden sensorineural hearing loss (SSNHL) is a deterioration of hearing greater than 30 dB occurring in at least three consecutive audiometric frequencies over a period of up to 72 h [22]. Idiopathic SSNHL affects 5 to 20 per 100,000 individuals with the probability of appearance increasing with age. This illness is considered to be a true otologic emergency [23]. For smaller losses, some patients regain their pre-loss hearing threshold without medical therapy due to the repair capacity of the cochlea, but in cases of profound hearing impairment the chance of complete recovery is limited [19]. The majority (about 90%) of SSNHL cases is idiopathic and several causes have been proposed to explain its etiopathogenesis (i.e., viral infection, otologic disease, trauma, vascular causes, and neoplastic disease, in order from the most frequent to rare [24]. Diagnosis and treatment should be preceded by a thorough medical history and physical examination of the patient. Adult-onset SSNHL typically affects women and men in the age between 43 and 53, with no significant gender differences. Patients typically present with tinnitus, vertigo, or ear fullness along with hearing loss. Tinnitus (a phantom auditory sensation without external sound, often described as a ringing, buzzing, or roaring in the ear) is the most frequent secondary symptom of idiopathic SSNHL; recent trials reported incidence rates as high as 73% to 84% [25]. The majority of sudden deafness cases are unilateral. Bilateral symptoms occur in less than 2% of all cases [26]. During the onset of SSNHL, the following variables are correlated with a worse prognosis: dizziness, profound hearing loss, impaired hearing in the contralateral ear, and delay to start treatment. Tinnitus at the onset of idiopathic SSNHL is reported to correlate with a better prognosis [27]. Idiopathic healing occurs in 32–65% of patients; the majority within the first 2 weeks after symptoms onset. Complete hearing recovery and full tinnitus remission were both reported about three times more frequent in mild–moderate hearing loss patients than in severe–profound cases [25].

The pathophysiology of SSNHL seems to be closely associated with the state of the inner ear. In the research of Gupta et al. [28], a low birth weight of infants (<5.5 lbs) was associated with higher risk of adult-onset hearing loss. The authors supposed that among the low birth-weight infants, exposure to malnutrition or stress may adversely affect cochlear development and influence the inner ear functioning in adult life. There is evidence suggesting that chronic inflammation may be involved in the development of idiopathic sensorineural deafness as a molecular basis of this illness [29,30,31]. It can lead to microvascular injury and atherogenesis and increase the risk of ischemia [32]. Inflammation can result in endothelial dysfunction, which can cause a thrombotic event that alters the blood supply to the inner ear [29]. The cochlea is an organ dependent on adequate oxygen levels in the blood [33]. However, because of the protected location of the cochlea in the temporal bone, blood supply to this organ is quite limited [34]. Blood is supplied to the cochlea mainly through a single terminal (labyrinthine) artery. Cochlear hair cells have a high oxygen consumption and poor tolerance of hypoxia, which is the reason why the inner ear is prone to changes in circulation. SSNHL appears to be characterized by hypoxia in the perilymph and, therefore, in the scala tympani and the organ of Corti. Thus, a mechanism playing a possible role in idiopathic SSNHL seems to be of vascular origin related to the lack of oxygen.

The prognosis in cases of isolated SSNHL is generally good, and an improvement within a matter of days is common. Patients in whom there is no change within 2 weeks are unlikely to show much recovery. However, the time between onset and treatment seems to be crucial in the prognosis. According to studies, 65% of patients with idiopathic SSNHL recovered their hearing irrespective of the type of treatment, with most recovering within 14 days [35].

Different therapeutic approaches are based on the supposed pathophysiological mechanisms responsible for inner ear dysfunction. Therapies of idiopathic SSNHL include corticosteroids, vasodilators, anticoagulants, antioxidants, plasma expanders and HBOT [36,37,38]. The first-line treatment for idiopathic SSNHL are oral or/and intratympanic corticosteroids to reduce the supposed inflammatory response. Steroids are one of the most used options among the therapeutic armamentarium without any strong recommendation to refer to. Oral steroids are usually proposed as a first-line treatment based on an evaluation of the ratio risk versus benefit [39]. The side effects expected from an acute therapy with oral steroids are mild [38]. However, trans-tympanic steroids are also recommended, because this mode of administration avoids the undesirable effects of systemic steroids. Trans-tympanic steroids in the treatment of SSNHL may be used as a primary therapy alone or in combination with systemic steroids, or as a salvage therapy after the failure of systemic steroids [38,39]. Research showed that intratympanic treatment was not inferior to oral corticosteroids treatment [38]. The success rate of the initial steroid therapy depends on many factors, such as the severity of the initial hearing loss and the time of the onset of treatment. In cases where the initial therapy of idiopathic SSNHL has no or an unsatisfactory result, adjunctive or salvage therapies are implemented. HBOT is mostly used as salvage therapy after the failure of the initial steroid therapy or as adjunctive therapy of idiopathic cases of sudden deafness treated with corticosteroids [2,22,37,39,40,41].

HBOT in the treatment of SSNHL is used to reverse the lack of oxygen in the inner ear. It increases the partial oxygen pressure to improve the blood oxygen profile and microcirculation. Oxygen supply is very important for hearing due to essential role of the endocochlear potential in auditory transduction in human. The cochlea mediates the transduction of sound waves into nerve impulses and this process depends on the endocochlear potential. Its magnitude is dependent on the oxygen supply. When the oxygen content of blood is increased, the magnitude of the endocochlear potential is elevated and auditory sensitivity is enhanced. The metabolic demand for the generation of endocochlear potential requires a dense capillary network for the delivery of the oxygen and removal of CO_2_. The lack of oxygen causes the lack of endovascular potential and results in sensorineural hearing loss. In a state of low oxygen tension, the endocochlear potential was found to be more negative than in the oxygenated state [19]. It was found that perilymphatic oxygen tension decreased in patients with SSNHL, while under hyperbaric conditions, the oxygen tension in the perilymphatic fluid increased by 450% [37]. According to the results of actual research, HBOT improves hearing in SSNHL patients by suppressing the inflammatory response induced by Toll-like receptors, which play a critical role in the inflammatory response [30]. These shed light on the molecular mechanism of the therapeutic effects of HBOT on hearing loss. By increasing the oxygen delivery to the cochlea, HBOT influence the endocochlear potential. HBOT seems to decrease the expression of inflammation-related cytokines in peripheral blood, leading to an improvement in the hearing levels in SSNHL patients [30,31].

HBOT was first used for SSNHL in the late 1970s [42]. Today oxygenation in hyperbaric conditions is still the only known method of increasing the oxygen level in the liquids of the inner ear. The purpose of HBOT in the treatment of SSNHL is to increase the partial pressure of oxygen in the blood and then, via diffusion, to increase the partial pressure of oxygen in the inner ear fluids that nourish the sensory and neural elements of the cochlea [19,20,42]. Oxygenation in hyperbaric conditions has gained popularity as a treatment for SSNHL in combination with pharmacologic agents. Recent evidence suggests that HBOT confers a significant additional therapeutic benefit when used in combination with steroid therapy for idiopathic sudden deafness. Results of recent research showed that HBOT combined with oral or intratympanic corticosteroids increased hearing gain in SSNHL patients compared to steroids treatment alone. According to the results of current research, when combined with a large-dose steroid treatment, HBOT gives positive results in 59.7–86.67% of patients with a greater rate of improvement (defined as gain of hearing of at least 10 dB on pure tone average) in patients treated with adjunctive hyperbaric oxygen than in patients treated only with corticosteroids [30,42,43,44,45,46,47,48]. In the majority of available research, the overall hearing recovery rate was significantly higher for the patients treated with hyperbaric oxygen and systemic steroids than for those treated with systemic steroids alone or systemic steroids combined with intratympanic steroids (Table 1).

There is only a small number of research that showed no significant differences between the steroid and steroid + HBOT groups in terms of hearing gain [48,50]; however, in some of these studies the treatment was implemented up to 30 days from the symptoms onset, which could influence the results of therapy. 

The actual results present no significant differences in therapeutic effect of HBOT alone in the treatment of SSNHL. In the research of Sun et al., corticosteroids showed a much better improvement of tinnitus than HBOT [51]. It stays in concordance with the actual EHMS recommendation for not using HBOT in the treatment of tinnitus due to a lack of a clear and confirmed clinical effect [2].

### 4.1. Implementation of Treatment 

In the available literature and guidelines regarding the treatment of SSNHL with the use of HBOT, special attention is paid to the time elapsed between the occurrence of symptoms and the commencement of treatment, with emphasis on the necessity of quick therapy implementation [2,19,40,41,47,49,52]. The effectiveness of HBOT is time-dependent and decreases with an increasing delay in administration. Generally, it is recommended to start with therapy as early as possible, preferably within 48 h [19]; however, there are discrepancies in the results of studies on the efficacy of HBOT treatment as a function of time. Research by Wang et al. [40] showed that the starting time of HBOT ≤ 7 days from disease onset was independently associated with hearing recovery. Capuano et al. found that recovery was significantly better when patients were treated with HBOT in the first 14 days after sudden deafness symptoms onset, compared to those treated after 14 days [49]. According to some reports, the rate of no recovery was significantly higher in patients who started treatment after 10 days of SSNHL onset [47]. Holy et al. found that patients who were treated with HBOT within 10days had significantly more improvement than those treated later than 10 days (66% vs. 39%). They describe treatment that started during the first 10 days following the onset of hearing loss as more effective than delayed treatment, even by only a few weeks [52]. Based on recent evidence, addition of HBOT to corticosteroids may be beneficial only when initiated early. The most favorable prognoses concern SSNHL patients with a medium or deep level of hearing loss (>41 dB), for whom HBOT was implemented within 14 days of the occurrence of the hearing loss [19,37,40,41,42,47,48,49,51,52,53]. According to the EHMS recommendation, it would be reasonable to use HBOT as an adjunct to corticosteroids in patients presenting after the first two weeks but not later than one month, particularly in patients with severe and profound hearing loss [2]. On the Consensus Conference on Hyperbaric Medicine in 2016, the EHMS recommended HBOT in the treatment of SSNHL as a Type 1 recommendation (therapy strongly recommended). However, the EHMS recommends HBOT combined with medical therapy only in patients with acute idiopathic SSNHL who present within two weeks from disease onset. According to EHMS, it would also be reasonable to use HBOT as an adjunct to corticosteroids in patients with severe or profound hearing loss (≥70 dB), but only in cases presented not later than one month after symptoms onset. There is no recommendation for HBOT in patients with SSNHL after six months of disease onset, due to the lack of randomized controlled clinical trials to confirm any positive effect of HBOT in such cases [2]. The guideline for clinical practice for idiopathic SSNHL, which was updated in 2019, states that HBOT can be administered as the initial therapy in combination with steroids within the first two weeks of symptoms onset, or as salvage therapy within the first month [54].

### 4.2. Severity of the Initial Hearing Loss 

In the light of current research, the addition of HBOT to primary conventional therapy improves the results when started early after the onset of SSNHL. The results of the treatment are also related to the severity of the initial hearing loss. In the research of Ajduk et al., a significant difference in the hearing thresholds after HBOT, used as salvation therapy after the failure of steroid therapy, was found at all frequencies in patients with a hearing loss of >61 dB. The group of patients with a hearing threshold of ≤60 dB had a significant improvement only at 250 and 500 Hz [37]. Similar results are reported by Hara et al. [55], who hypothesized that HBOT improves hearing in idiopathic SSNHL patients at lower frequencies. Using early HBOT as adjunctive therapy to pharmacological treatment in many research studies showed the most promising therapeutic effects among patients with severe/profound (≥70 dB) and complete hearing loss [19,39,40,42,47,51,55,56,57]. Hosokawa et al. [44] found significantly better improvement rates in patients who were 60 years old or younger (74.8%) compared to patients older than 60 years (62.9%). Current research demonstrated also that a lower proportion of men was associated with a greater benefit of HBOT in comparison to women [56]. Early-stage combination therapy for patients in many research studies was associated with better audiometric results at higher frequencies and a better clinical outcome score [58].

### 4.3. HBOT Strategy 

For HBOT to be properly effective in the treatment of SSNHL, the pressure in the hyperbaric chamber during HBOT should be at least 1.4 ATA. Only breathing 100% oxygen in hyperbaric conditions above 1.4 ATA contributes to a significant increase in the diffusion radius of the oxygen from the capillaries to the surrounding tissues [4,5,6]. Contemporary research show that increasing the maximal pressure during HBOT higher than 2.5 ATA did not provide any benefit for hearing recovery; so, an air pressure kept at 2.0 to 2.5 ATA is currently recommended for the treatment of SSNHL [56]. In terms of HBOT strategy, the optimal number of sessions in the treatment of sudden deafness should be determined individually for each case; however, there is no evidence in the literature for the effectiveness of HBOT longer than 10–20 sessions [59]. The lowest effective number of HBOT sessions should be implemented. Research reported a positive therapeutic effect of HBOT at 2.5 ATA in a 2-week protocol with a 90-min session per day, applied together with pharmacological treatment [41,55,56,59,60]. In a report of Olex-Zarychta [41], a full hearing recovery after 15 daily HBOT sessions was observed, with hearing gain reported by the patient after six sessions. In the research of Rhee et al. [56], complete hearing recovery was favored in SSNHL patients with a total HBOT duration of at least 1200 min. The authors recommended that 100% oxygen at 2.0 to 2.5 ATA should be administered for 10 to 20 days, with a 90-min session each day. Hara et al. [55] presented a protocol consisting of HBOT for 20 sessions, together with pharmacotherapy once a day for seven consecutive days, performed 3 h before the HBOT session. Generally, early implementation of a 10-day protocol with a 90-min session per day is recommended, or, alternatively, a 20-day protocol with a 60-min session per day to achieve a total HBOT duration of at least 1200 min. A medical consultation by a specialist in hyperbaric medicine before starting HBOT is recommended [41]. According to ECHM guidelines, a treatment series of HBOT in the treatment of SSNHL should consist of 15 daily 60-min exposures to 100% oxygen at 2.5 atmosphere ATA. All sessions should take place at the same time every day and at the same place (professional mono-place or multi-place hyperbaric chamber). According to official ECHM recommendations, each session of HBOT should last 90 min and should be divided into several phases: 10 min for air compression, 70 min of therapy (3 × 20 min of breathing pure oxygen through the mask with two 5-min breaks with the mask off), and about 10 min for decompression [2,41]. 

## 5. Adverse Reactions to HBOT

The use of oxygenation in hyperbaric conditions as a medical procedure is associated with the possibility of side effects and complications. HBOT remains among the safest therapies used today; however, some side effects are associated with this procedure [61,62,63,64,65]. HBOT side effects are primarily the result of increased pressure or hyperoxia. Possible complications during HBOT include barotraumatic lesions (middle ear, nasal sinuses, inner ear, lung, and teeth), oxygen toxicity (central nervous system, and lung), confinement anxiety (claustrophobia), and ocular effects (myopia and cataract growth) [2,6,7,61,62,63,64,65]. Undesirable reactions may occur during and after exposure to 100% oxygen, and its toxicity must always be taken into consideration before the implementation of HBOT in the treatment of SSNHL.

### 5.1. Effects of Pressure

The predominant complication is represented by pressure equalization problems within the middle ear that occur during the compression phase of the HBOT session. The most frequent complication of HBOT is barotrauma; it may affect the middle ear, but also the accessory sinuses of the nose and lungs. For example, 15–43% of patients experience symptoms of barotrauma in the middle ear during the compression phase of the HBOT procedure [2,7,61,62,63,64]. They have ear ache or problems with unblocking the hearing tube to relieve pressure. Recent publications indicated a vast majority (84%) of barotrauma complications being minor trauma (tympanic membrane injection or its slight hemorrhage) and no cases of tympanic membrane rupture. Risk factors that have been identified include a very slow or high rate of compression, intubation, acute upper respiratory infection, and a history of head and neck malignancy [64]. In order to prevent pressure injuries, pharmacologic agents are administered either orally or intranasally, and paracentesis can also be performed. In some cases, the procedure of compression must be interrupted, and the patient must leave the hyperbaric chamber. A pressure shock may occur on a one-off basis and often affects only one ear. To minimalize the risk of pressure injuries, the compression and decompression phases of the HBOT protocol are recommended to be done very slowly [2,7]. Otherwise, edema in the middle ear may occur as well as retraction or even perforation of the tympanic membrane, which may lead to hearing deficits. In rare cases, pressure trauma to the middle ear can damage the inner ear and impair its function. There is a risk of rupture of the oval or round window membranes [61,63].

### 5.2. Pulmonary Oxygen Toxicity

Pulmonary oxygen toxicity is not expected from routine daily HBOT when used for typical elective indications, but it is a possible side effect of oxygenation in hyperbaric conditions [64]. Pulmonary toxicity from the oxygen is associated with long-term exposure of the body to 100% oxygen under both normal and elevated pressure conditions. An oxygen toxicity seizure is relatively rare at typical clinical treatment pressures (2–3 ATA). It is difficult to predict on an individual basis. It was traditionally reported at ~1 in 10,000 treatments [61]. Early symptoms of oxygen poisoning are irritation of the larynx and trachea, periodontal pain of the larynx, and swelling of the nasal mucosa. Such symptoms may appear after approx. 6 h of breathing with 100% oxygen at 2 ATA. Oxygen delivery by the intermittent method and a limited time of exposure (about 60–70 min) are practical ways to avoid symptoms of pulmonary oxygen toxicity under hyperbaric conditions. 

### 5.3. Ocular Side Effects

Eye complications related to HBOT include short-sightedness (myopia) and cataracts. Myopia is a transient condition occurring in about 20–100% of patients undergoing oxygen therapy in hyperbaric chambers [61,62,63]. Progressive myopic changes to the eye may occur after repetitive HBOT. The cause of periodic visual impairment related to HBO intervention is not clear. The exact mechanism is not fully understood, but it is thought that oxygen toxicity causes changes to the crystalline lens, hardening the lens and increasing its refractive power. It is believed that temporary visual impairment can be inter alia because of changes in the curvature of the cornea caused by variations in pressure during the compression and/or decompression, metabolic changes in the cornea, or changes in the refractive lens [6]. Research also showed a possible increase in the risk of irreversible refractive changes if the number of therapies exceeds 100 [66]. Most of the myopic changes are reversible after cessation of the therapy. Many studies report this effect to last from days to months. Myopia is a reversible effect; in the majority of cases, the pre-therapy level of vision is reached within 6–8 weeks of HBOT completion [61,64]. However, in some reports the time for eyesight to normalize after HBOT was reported to be different. In a study by Olex-Zarychta, the reversal of myopia was rapid, within the first ten days after the last HBOT session. In this particular case the therapy included only 15 HBOT exposures [41]. However, reversal of myopia may progress more slowly over months up to a year from the date of completion of HBOT [6]. 

An excess of reactive oxygen species in tissues due to their cytotoxic properties and/or deficiencies in antioxidant activity may contribute to complications of HBOT, such as cataracts. The exact mechanism of cataractogenesis is not completely understood. The pathogenetic processes in the keratoconus seem to be contributing by deficiencies in antioxidant activities and reactive oxygen species. It is supposed that such an impact may be exacerbated during HBOT by exposure to additional reactive oxygen species [62]. The development of both nuclear cataracts and a reversible myopic shift in hyperbaric patients strongly suggests that oxidative damage to the lens proteins is responsible for these ocular complications of HBOT [66].

Limited exposure to HBOT (no more than 20–60 treatment sessions) does not seem to cause cataracts. According to reports, cataract formation may occur only in a long treatment series greatly exceeding 60 sessions. However, HBOT can lead to more rapid progression of existing cataracts [61,64,66,67]. In the literature, the role of the age of the patient is emphasized. Cataracts remain the leading cause of visual impairment and blindness worldwide and usually occurs after the age of 65 [61]. The senescent eye is supposed to be particularly prone to oxidative damage, exemplified by cataract and macular degeneration. The retina is particularly susceptible to oxidative stress, because of its high consumption of the oxygen. Age-related macular degeneration involves oxidative stress and death of the retinal pigment epithelial cells. HBOT may exacerbate these processes. That is the reason why cataracts as well as age-related macular degeneration and keratoconus may be contraindications to HBOT and exposure to the therapy-related oxidative stress. In the study by Palmquist et al. [66], over 40% of patients with no evidence of a cataract before therapy developed nuclear cataracts, with the first evidence appearing after 150 HBOT sessions. Long-term exposure to hyperbaric oxygenation, resulting in oxidative damage to the lens proteins, seems to play a critical role in this ocular complication of HBOT [61,66,67]. It should be underlined that ocular changes are not fully reversible after cessation of prolonged HBOT. Careful pre-examination and evaluation of the potential risks and benefits from HBOT should be warranted to the patient. Delivery of HBOT may need to be modified or it may even be contraindicated in some cases [61,62,67]. Patients who undergo multiple HBOT treatments and complain of visual changes persisting for more than two to three months after completion of the HBOT course should be seen by an ophthalmologist to assess their vision and obtain a diagnosis of the cause of any impairment [67].

### 5.4. Central Nervous System Oxygen Toxicity

The effects of HBOT on neural elements remain poorly understood [65]. The toxic effect of oxygen on the central nervous system (CNS) can manifest itself after even a short breathing time of 100% oxygen at an elevated pressure (at least 2 ATA). The recognized presentation of CNS oxygen toxicity during clinical hyperbaric oxygen treatment is an oxygen toxicity seizure. Recent evidence over the past 15 years puts the incidence at ~1 in 2000–10,000 treatments [61,66]. HBOT-induced seizures are defined as brief, oxygen-related, generalized tonic–clonic convulsions usually occurring toward the end of the treatment [68]. The higher the compression pressure, the faster the clinical symptoms of the toxic effects of oxygen on the CNS may appear [63]. The most characteristic symptom of CNS oxygen toxicity is generalized grand mal convulsions. The seizure may be preceded by muscular tremors around the mouth, eyes, and by trembling hands [6,7,61,65]. HBOT seems to be a safe method for patients with neurological disorders, excluding those who suffer from uncontrolled epilepsy [68]. Cerebral toxicity of the oxygen may be avoided by strict adherence to medical procedures for HBO treatment, including intermittent and controlled oxygen administration in hyperbaric chambers. The reason for the increased incidence for CNS oxygen toxicity during HBOT appears to be related to patient selection and changes in hyperbaric oxygen treatment protocols [61,68]. 

## 6. Conclusions

SSNHL appears to be characterized by hypoxia in the perilymph in the scala tympani and the organ of Corti. It is supposed that a possible mechanism of idiopathic SSNHL is of vascular origin and related to the lack of oxygen. HBOT is still the only known method of increasing the oxygen level in the liquids of the inner ear. Due to limited adverse reactions and compelling positive outcomes stemming from HBOT in the literature, this procedure is recommended clinically as adjunctive therapy for the treatment of SSNHL. Hyperbaric oxygen therapy remains an option, but only when combined with steroid therapy for either initial treatment or salvage therapy after the failure of pharmacological treatment. The high effectiveness of early HBOT implementation together with standard pharmacological treatment has been proven clinically and from 2016 is strongly recommended by EHMS for patients with acute idiopathic sudden deafness who present within two weeks of disease onset, or as a salvage therapy implemented within 1 month of onset of sensorineural hearing loss. The optimal number of HBOT sessions in the treatment of SSNHL depends on the severity and duration of the symptoms and on the response to the treatment. Standard treatment includes on average 10/20 daily sessions making a total HBOT duration of at least 1200 min. The typical time of one HBOT session recommended by EHMS is 90 min divided into three main phases; the time of breathing pure oxygen in the main phase of the session is about 60 min. Delivery of hyperbaric oxygenation may need to be modified or contraindicated in individual cases due to possible complications. Patients scheduled for HBOT need a careful pre-examination and monitoring. If safety guidelines are strictly followed, HBOT is a treatment method with a relatively low rate of adverse reactions.

## Figures and Tables

**Table 1:** The percentage of hearing gain and recovery in comparative studies of hyperbaric oxygen therapy (HBOT) and pharmacotherapy with corticosteroids (CS) in sudden sensorineural hearing loss (SSNHL).

Author	Year	Percentage of Hearing Gain, *p* < 0.005	
		CS	CS + HBOT
Satar et al. [48]	2006	76.4%	60.0% *
Fujimura et al. [42]	2007	39.7%	59.7%
Capuano et al. [49]	2015	68%	84%
Cho et al. [46]	2018	40.8%	65.7%
Krajcovicova et al. [45]	2018	28.6%	61.7%
Yücel and Özbuğday [50]	2020	60%	68.2% *
Liu et al. [30]	2020	66.67%	86.67%

* Result not significant.

## Abbreviations

ATA	atmosphere absolute
ATP	adenosine-triphosphate
CNS	central nervous system
CO	carbon monoxide
CO_2_	carbon dioxide
ECHM	European Committee of Hyperbaric Medicine
HBOT	hyperbaric oxygen therapy
NO	nitric oxide
SSNHL	sudden sensorineural hearing loss
UHMS	Undersea and Hyperbaric Medical Society
WHO	World Health Organization
